# Dengue Prevention and 35 Years of Vector Control in Singapore

**DOI:** 10.3201/10.3201/eid1206.051210

**Published:** 2006-06

**Authors:** Eng-Eong Ooi, Kee-Tai Goh, Duane J. Gubler

**Affiliations:** * DSO National Laboratories, Singapore;; † Ministry of Health, Singapore;; ‡ University of Hawaii at Manoa, Honolulu, Hawaii, USA

**Keywords:** Dengue, prevention, vector control, surveillance, synopsis

## Abstract

After a 15-year period of low incidence, dengue has reemerged in Singapore in the past decade. We identify potential causes of this resurgence. A combination of lowered herd immunity, virus transmission outside the home, an increase in the age of infection, and the adoption of a case-reactive approach to vector control contribute to the increased dengue incidence. Singapore's experience with dengue indicates that prevention efforts may not be sustainable. For renewed success, Singapore needs to return to a vector control program that is based on carefully collected entomologic and epidemiologic data. Singapore's taking on a leadership role in strengthening disease surveillance and control in Southeast Asia may also be useful in reducing virus importation.

## Abstract 2

A vector control program must be based on epidemiologic and entomologic data.

Dengue fever (DF) and dengue hemorrhagic fever (DHF) are reemerging diseases that are endemic in the tropical world. Disease is caused by 4 closely related dengue viruses that belong to the genus *Flavivirus* and are transmitted principally by the *Aedes aegypti* mosquito. Other mosquito species, such as *A. albopictus* and *A. polynesiensis*, can transmit epidemic dengue but do so less efficiently (*1*). The virus has 4 antigenically similar but immunologically distinct serotypes. Infection confers lifelong immunity to the infecting serotype but not to the remaining 3; therefore, a person can be infected with dengue virus up to 4 times during his or her lifetime. Furthermore, epidemiologic observations suggest that previous infection increases risk for DHF and dengue shock syndrome (DSS) in subsequent infections (*2*). These conditions are characterized by plasma leakage as a result of alteration in microvascular permeability (*3*). While DF may cause substantial morbidity, the death ratio of DHF and DSS can be as high as 30% if the disease is not properly managed (*4*). As yet, no specific treatment for DF or DHF is available, although efforts to develop an antidengue drug are in progress.

While vaccines for other flaviviruses such as yellow fever and Japanese encephalitis have been developed, dengue vaccine development is complicated by the need to incorporate all 4 virus serotypes into a single preparation. An approved vaccine is not likely to be available for 5 to 7 years; the only way to prevent dengue transmission, therefore, is to reduce the population of its principal vector, *A. aegypti*.

Dengue has been successfully prevented through vector control in 3 instances. The first of these was the highly successful, vertically structured paramilitary hemispheric eradication campaign directed by the Pan American Sanitary Board from 1946 to 1970 (*5*). The second was also a rigorous, top-down, military-like vector control operation in Cuba that was based on intensive insecticidal treatment followed by reduction of available larval habitats (source reduction) in 1981 (*6*). Neither of these programs, however, was sustainable. The third successful program was in Singapore.

## Vector Control in Singapore

DHF appeared in Singapore in the 1960s and quickly became a major cause of childhood death. Public health response to dengue began in 1966, when the Vector Control Unit was set up within the Quarantine and Epidemiology Branch, initially in the Ministry of Health but transferred to the Ministry of the Environment in 1972, when DHF was made a notifiable disease (*7*); DF was made notifiable in 1977. From 1966 to 1968, following a series of entomologic surveys (*8**–**12*) and a pilot project to control the *Aedes* vectors in an area with high incidence of DHF (*13*), a vector control system based on entomologic surveillance and larval source reduction (i.e., reducing the availability of *Aedes* larval habitats) was developed; the system was implemented in 1968 (*7*). The thrust of this program was that mosquito breeding precedes disease transmission and controlling the vector population before disease is detected would reduce transmission. In a pilot project, this approach reduced the *A. aegypti* population in a 3-month period from 16% to 2%, as measured by the premises index, which is the percentage of inspected premises found to have containers with *A. aegypti* larvae or pupae (*13*). To maintain this low vector population density, however, the pilot study concluded that public involvement was necessary because the vector repopulates the area soon after vector control operations move to another site (*13*). The vector control program thus has 2 elements in addition to source reduction: public education and law enforcement. The Destruction of Disease Bearing Insects Act of 1968 was enacted to discourage persons from intentionally or unintentionally propagating mosquitoes.

**Figure 1:**
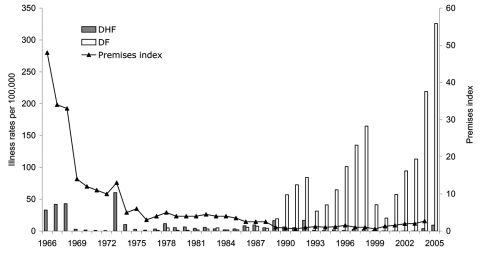
Annual incidence dengue fever (DF) and dengue hemorrhagic fever (DHF) and the premises index, Singapore, 1966–2005. DHF was made a notifiable disease in 1966, while DF became a notifiable disease in 1977. The annual incidences of DF and DHF reported in this figure were calculated from the number of reported cases each year from 1966 to 2004. The annual premises index is expressed as a percentage of the premises in which *Aedes aegypti* or *A. albopictus* larvae were found divided by the number of premises visited by environmental health officers.

The implementation of this vector control program was completed in 1973. The premises index since then has been ≈2%; achieving an index of zero has been difficult since natural breeding habitats are created as quickly as they are eliminated (*14*). With the reduced *A. aegypti* population, Singapore experienced a 15-year period of low dengue incidence. However, since the 1990s, the incidence of dengue has surged despite the low premises index (Figure 1).

Singapore's experience with dengue bodes ill for the sustainability of preventive efforts. Vector control may have worsened the dengue situation in Singapore because overt dengue attack rates in the 1990s and early 2000s were severalfold higher than those in the 1960s. We have identified several factors that may have contributed to this resurgence: lowered herd immunity, increasing virus transmission outside the home, more clinically overt infection as a consequence of adult infection, and a shift in the surveillance emphasis of the vector control program. We discuss each of these factors and suggest possible solutions.

## Lowered Herd Immunity

Several explanations have been put forth to account for the resurgence of dengue in Singapore despite the vector control program (Figure 1). Reduced dengue transmission in the 1970s and 1980s resulted in a concomitant reduction in herd immunity to dengue virus (*15*). Low levels of population immunity provide an ideal condition for dengue transmission despite low *Aedes* mosquito density (*16*). This hypothesis is supported by observations made from a series of serologic surveys conducted in 1982–1984, 1990–1991, and 1993, in which a declining trend of seroprevalence among children was observed (*17*).

Low herd immunity in the Singapore population could also be deduced by comparing seroprevalence ratios with those of other dengue-endemic countries. The seropositive ratios of 6.7% in primary school children and 42% in adults (*18*) are in contrast to ratios reported in other dengue-endemic countries such as Thailand, where primary school children in Ratchaburi Province had a seropositive rate of 71% (*19*).

## Transmission Outside the Home

Lowered herd immunity is, however, insufficient to account for the resurgence of dengue in Singapore. Dengue is predominantly a childhood disease in most parts of Southeast Asia, and more women than men are infected as adults. This disease pattern fits the behavior of *A. aegypti*. This species of mosquito is highly domesticated, lives and breeds indoors, has a limited flight range, and feeds almost exclusively on humans. Consequently, persons who spend more time at home during the daytime, i.e., mothers and children, are more likely to be infected than those who leave the home for work. In Singapore, however, the incidence of DF/DHF is lower in children than in adults (*14*). This finding could be due to a high proportion of subclinical infection in children or a lack of infection in the domestic environment.

To investigate this observation, a serologic survey of 1,068 children <15 years of age was conducted during an 18-month period in 1996 and 1997 (*17*). All children who were born at or who visited outpatient clinics of the National University Hospital, which serves the entire country, for routine check ups and vaccinations were included in this study, with parental consent. This population would have grown up during dengue resurgence. The results of this survey showed that preschool children, 10 months to 5 years of age, had a seroprevalence ratio of 0.77%, children 6–10 years of age and 11–15 years of age has prevalence ratios of 6.7% and 6.5%, respectively (*17*). School-age children were therefore 9× more likely to have antibodies to dengue than were preschool children (*17*).

Preschool children spend most of their time either at home or at a nursery or kindergarten. Most of these facilities are run out of residences or shophouses in government-owned, high-rise accommodations. Formal half-day schooling starts at the age of 6 years, often with after-school extracurricular activities. The significant difference in seropositivity between preschool and school-age children suggests that the risk of acquiring dengue in Singapore is greater when a person spends more time away from home (*17*).

This hypothesis is supported by the lower premises index in residences than nonresidences in 1997. Residential properties in 1997 had low premises indexes; 2.1% in landed premises and 0.6% in apartments compared to indexes in schools (27.0%), construction sites (8.3%), factories (7.8%), and vacant properties (14.6%) (*20*). In contrast, the premises index in 1966 was highest in residences: slum housing (27.2%), shophouses (16.4%), and apartments (5.0%) (*9*). Furthermore, women, who are more likely than men to care for children at home, have a lower incidence of dengue, as indicated by the male-to-female disease ratio of 1.6:1 (*21*). Collectively, these findings suggest that substantial virus transmission occurs away from the home.

## Dengue in Adults

**Figure 2:**
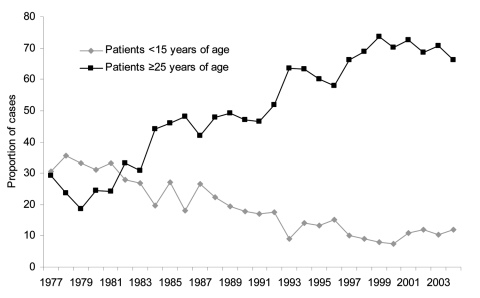
Proportion of indigenous dengue fever cases in patients <15 or >25 years of age, Singapore, 1977–2004. Indigenous cases are those that were acquired locally, among permanent and temporary residents of Singapore. Data were obtained from Communicable Disease Surveillance in Singapore, an annual publication of the Ministry of the Environment until 2002 and the Ministry of Health since 2003.

As a consequence of lowered herd immunity and transmission outside the home, cases in adults predominate in Singapore. This fact is reflected in the steady decline in the proportion of patients <15 years of age, while the proportion of patients >25 years of age has increased over the years (Figure 2). This predominance of cases in adults may also contribute to the resurgence in dengue incidence. While most dengue infections, particularly primary infections in young children, are mild or silent (*22**,**23*), infections in adults are more likely to be clinically overt. In a recent dengue fever outbreak at a construction site in Singapore, patients had serologic and virologic evidence of primary dengue infection with serotype 2 virus (*24*). A serologic survey was conducted in the affected construction site; 274 of 360 workers volunteered for the study. With anti-dengue immunoglobulin M used as a marker, the survey identified 27 workers with recent infection. The illness was sufficiently debilitating for 24 (88.9%) of them to seek medical attention (*24*). Results support the commonly held perception that dengue infection in adults is more likely to be clinically overt than in children, contributing to the increase in the overall incidence of dengue.

A second consequence of the increase in patient age may be in the outcome of dengue infection. With the increase in patient age, most dengue cases in Singapore manifest as DF instead of DHF (Figure 1), even though a substantial proportion of adults have neutralizing antibodies to >2 serotypes of the dengue virus (*25*). The observed epidemiologic trend in Singapore therefore suggests that adults, while still susceptible, are at lower risk for DHF than are children. In the 1981 dengue outbreak in Cuba, hospitalization and death rates for severe and very severe dengue, postulated to be equivalent to DHF and DSS, were highest in those <15 years of age and those >60 years of age (*26*). Hospitalization and death rates were lower for those whose ages fell between these age groups, despite the same secondary infection with dengue serotype 2 virus (*26*). Results from Cuba support the hypothesis that adults are at lower risk for DHF and DSS than are children.

These age-dependent differences in the outcome of dengue infection may be due to differences in vascular permeability; children have a greater propensity for vascular leakage, under normal physiologic conditions, than do adults (*27*). This higher baseline of microvascular permeability in children could result in less ability to accommodate extraneous factors, such as dengue infection, that increase vascular permeability (*27*).

While the risk for DHF in adults is low compared to that in children, it is not absent. Besides host factors and secondary infection, certain strains of dengue viruses have been associated with severe disease (*28**,**29*). Although more work is needed to elucidate the role that age and other host and viral factors play in the pathogenesis of DHF, the current low DHF incidence cannot be taken as invulnerability to DHF outbreaks.

## Shift in Surveillance Emphasis

Without a vaccine or antiviral drug, an effective vector control program is the only means to reduce dengue transmission. While most components of the vector control program remain similar to those of the 1970s, differences exist. Over time, the program evolved and its strategy changed. In particular, emphasis is now placed on early detection of cases and identifying whether they cluster in time and space, which is taken to indicate active virus transmission in the area. Detecting such clusters triggers emergency vector control operations, as was observed during a recent review of dengue in Singapore (*30*).

This shift of emphasis away from vector surveillance toward case detection cannot be linked with certainty to specific factors or events. Previous reviews of the dengue control program in Singapore in 1993 (*31*), 1994 (*32*), and 1997 (*33*) made the same observation. The shift probably took place in the late 1980s or early 1990s since Chan's report on the program in 1985 continued to emphasize vector surveillance (*7*). The shift in emphasis coincides with the latter stages of the 15-year period of low dengue incidence. With vector control, dengue transmission may have become sporadic and isolated, making perifocal mosquito control in response to reported cases more widely practiced as an efficient means of using public resources. Entomologic surveillance-based vector control still exists but only in limited, dengue-sensitive areas (*31**–**33*).

Responding to dengue cases and clusters, however, has limited effectiveness in preventing virus transmission, since such an approach ignores virus transmission from persons with subclinical infection or mild undifferentiated fever to uninfected mosquitoes. Furthermore, only ≈30% of cases can be mapped to a cluster. Most reported dengue cases occur outside known clusters. No evidence shows that emergency control measures, particularly the use of chemical insecticides, are effective after cases have already been detected (*31*).

## Solutions

Observations made on epidemiologic features of dengue in Singapore indicate that further studies on the exact sites of dengue transmission need to be conducted. While the serologic study in children and the increasing proportion of adult infections, particularly among men, suggest that transmission may occur outside the home, further epidemiologic and virologic studies are needed. An ongoing case-control study, combining virus isolation, serotype identification, and genetic characterization of the virus by genome sequence analysis, may prove useful. Shedding more light on virus transmission dynamics would guide use of public health resources.

In addition to epidemiologic studies, more detailed entomologic research is needed. Larval source reduction and control are the most effective methods to deal with the *Aedes* vector. With lowered herd immunity and the possibility of virus transmission in nondomestic places, the vector control program in Singapore must return to an approach that emphasizes vector surveillance instead of early case detection. A repeat of some of the entomologic studies that were performed from 1966 to 1968 (*8**–**12*) may be fruitful. Results from such studies could help in revising the current vector control strategy and devising effective systems for surveillance of *A. aegypti*. Research on vector bionomics and evaluation of the cost-effectiveness of various control strategies may also be rewarding. Alternative approaches that complement larval source reduction should also be considered. We suggest 2 such approaches.

### Ovitraps

Public involvement in Singapore is crucial to the sustainability of a vector control program (*7**,**13*). Public education is therefore essential for Singapore's vector control effort. National campaigns, such as the month-long "Keep Singapore Clean and Mosquito Free" campaign in 1969, have been conducted, and schoolchildren have been educated to carry out source reduction in their homes. However, 2 community-based surveys in 1992 and 1995 showed that while the population's awareness of the need for dengue control is high, many respondents did not believe that mosquitoes were in their homes and did not carry out necessary preventive measures (*34*). The survey population also reported that they checked their homes for mosquito breeding after having been fined under the Destruction of Disease-Bearing Insects Act, which has been superseded by the 1998 Control of Vectors and Pesticide Act. The problem with threatening the public with legal repercussions is that in the absence of checks by vector control officers, the public is not motivated to prevent mosquito breeding. What may be necessary would be a method of engaging the public through tools that provide regular positive feedback to the users. The recent positive experience in Vietnam is a case in point (*35*). Members of the public were closely engaged in the vector control effort by cleaning public areas and using copepods in water storage tanks. While the water supply system in Singapore is vastly different from that in Vietnam, the principle of engaging the public with an effective larvicidal tool could be adopted.

**Figure 3:**
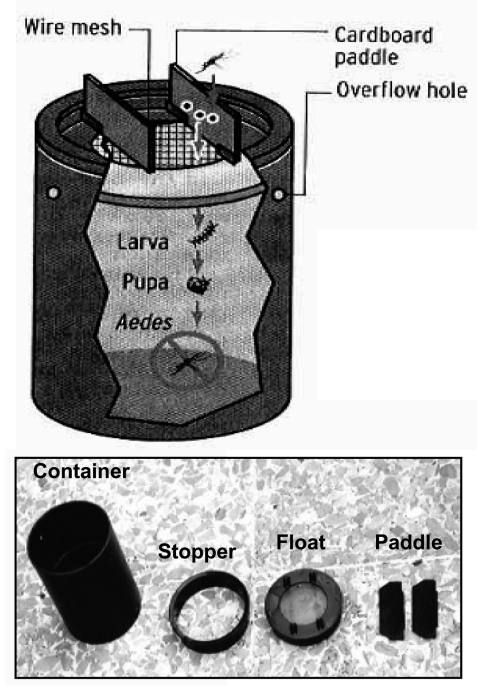
Diagrammatic representation of an autocidal ovitrap made up of a black cylinder, a wire mesh on a flotation device, and 2 pieces of cardboard. The gravid female *Aedes* mosquito lays its eggs on the cardboard. The larvae that hatch from the eggs go through the immature stages of the mosquito's lifecycle, but the resultant adult mosquito will be trapped underneath the wire mesh and drown. Picture inset shows the different components of the ovitrap.

A larvicidal ovitrap was introduced by Lok et al. (*36*) that consists of a black, water-filled cylindrical container with a flotation device made up of a wire mesh and 2 wooden paddles. Eggs laid by mosquitoes on the wooden paddle hatch, and larvae develop in the water under the wire mesh. Resultant adult mosquitoes are trapped under the wire mesh and drown (Figure 3).

The use of such an ovitrap has, in 2 previous instances, significantly reduced the *A. aegypti* population (*37**,**38*). The drawback in both these instances was that a large number of vector control officers were required to inspect and maintain the ovitraps. We suggest that such a trap could be used and maintained by members of the public instead of limiting its use to public employees. While training in the use and maintenance of ovitraps may be needed, seeing trapped mosquitoes may provide both positive feedback and a regular reminder of the need to be vigilant in efforts to curb the growth of the mosquito population.

An alternative to this ovitrap is a gravid female mosquito trap recently developed by Liew and Giger (patent pending). This device makes use of nondrying glue to trap gravid female mosquitoes that are attracted to water in the cylindrical device. In cage studies, this device maintained its effectiveness for up to 9 months (C. Liew, pers. comm.). Coupled with public education, using ovitraps or gravid female traps may sustain vector control more effectively than law enforcement.

### Strengthening Regional Vector Control

A bolder, but possibly more rewarding, approach would be for Singapore to take the lead in strengthening disease surveillance and vector control in the Southeast Asian region, where dengue remains hyperendemic. The constant importation of dengue virus by travelers to Singapore may contribute to the observed dengue resurgence (*39**,**40*). Each year, 8 million visitors arrive in Singapore, not including residents who travel abroad or the thousands who commute across the causeway from the southern peninsula of Malaysia. The Singapore Changi Airport alone handles >20 million passengers per year, a rate that might better illustrate the amount of human traffic through Singapore. In the past 5 years, 5%–10% of dengue cases in Singapore have been imported. Most of these cases are from Indonesia, Thailand, and Malaysia. Collectively, these data suggest that symptomatic and asymptomatic persons can easily enter Singapore and infect vector mosquitoes. This problem will continue to expand, since travel and trade in the region are likely to increase. Expanding resources and effort toward achieving vector control in Southeast Asian countries may reduce importation of dengue and overall dengue incidence in Singapore.

Indeed, the mechanism to facilitate regional cooperation for disease surveillance and control is already being established. The Regional Emerging Disease Intervention Centre officially opened in Singapore on May 24, 2004. A joint United States–Singapore collaboration, its mission involves extending the perimeter of defense for emerging infectious diseases, widening the international network for research, and translating research findings into improved public health. While its immediate focus is on avian influenza and the threat of a pandemic, dengue could become a key item on its agenda, and a regional, surveillance-based vector control effort could be initiated.

## Conclusions

In the absence of a safe and effective tetravalent vaccine for dengue viruses, vector control is the only method to prevent viral disease. The main lesson learned from Singapore's experience is that for a vector control program to be effective, it must be based on carefully collected and analyzed epidemiologic and entomologic surveillance data, with particular emphasis on ecologic factors that determine where, how, and when to initiate vector control, which Chan termed "vector epidemiology" (*7*). Reacting to cases, despite early and rapid diagnosis, is unlikely to reduce the incidence of dengue. An effective vector control program will require an increase in expenditures, new strategies to lower and limit the *A. aegypti* population, and limiting importation of dengue virus into Singapore.

The Singapore experience also underscores the fact that dengue control must be a regional effort. Barring eradication of the mosquito vector, countries that control dengue transmission are doomed to failure if neighboring countries do nothing to prevent continued epidemic transmission. Thus, the combination of decreasing herd immunity and increasing imported dengue infection make preventing dengue transmission difficult, even with *A. aegypti* indexes as low as 2%, as exists in Singapore.

A final justification for regional *A. aegypti* control in Southeast Asia is the potential for epidemic urban yellow fever in the American tropics, risk for which is at its highest level in 60 years. With modern transportation, urban yellow fever could move quickly from the American tropics to the Asia-Pacific region, where ≈2 billion people are at risk. While a safe, effective vaccine for yellow fever is available, it is not manufactured in large enough quantities to prevent or control epidemics in Asia. Thus, regional *A. aegypti* control would be an effective preventive measure for epidemic dengue and yellow fever in Asia.
